# Socioeconomic patterns of smoking cessation behavior in low and middle-income countries: Emerging evidence from the Global Adult Tobacco Surveys and International Tobacco Control Surveys

**DOI:** 10.1371/journal.pone.0220223

**Published:** 2019-09-06

**Authors:** Nigar Nargis, Hua-Hie Yong, Pete Driezen, Lazarous Mbulo, Luhua Zhao, Geoffrey T. Fong, Mary E. Thompson, Ron Borland, Krishna M. Palipudi, Gary A. Giovino, James F. Thrasher, Mohammad Siahpush

**Affiliations:** 1 American Cancer Society, Atlanta, Georgia, United States of America; 2 School of Psychology, Deakin University, Geelong, Victoria, Australia; 3 Department of Psychology, University of Waterloo, Waterloo, Ontario, Canada; 4 School of Public Health and Health Systems, University of Waterloo, Waterloo, Ontario, Canada; 5 Centers for Disease Control and Prevention, Atlanta, Georgia, United States of America; 6 CDC Foundation, Atlanta, Georgia, United States of America; 7 Ontario Institute for Cancer Research, Toronto, Ontario, Canada; 8 Department of Statistics and Actuarial Science, University of Waterloo, Waterloo, Ontario, Canada; 9 School of Psychological Sciences, University of Melbourne, Melbourne, Australia; 10 VicHealth Centre for Tobacco Control, Cancer Council Victoria, Melbourne, Victoria, Australia; 11 School of Public Health and Health Professions, University at Buffalo, SUNY, Buffalo, New York, United States of America; 12 Department of Health Promotion, Education, and Behavior, Arnold School of Public Health, University of South Carolina, Columbia, South Carolina, United States; 13 Departamento de Investigación sobre Tabaco, Instituto Nacional de Salud Pública, Cuernavaca, México; 14 Department of Health Promotion, Social & Behavioral Health, College of Public Health, University of Nebraska Medical Center, Nebraska, United States of America; Public Health Foundation of India, INDIA

## Abstract

**Introduction:**

Tobacco smoking is often more prevalent among those with lower socio-economic status (SES) in high-income countries, which can be driven by the inequalities in initiation and cessation of smoking. Smoking is a leading contributor to socio-economic disparities in health. To date, the evidence for any socio-economic inequality in smoking cessation is lacking, especially in low- and middle-income countries (LMICs). This study examined the association between cessation behaviours and SES of smokers from eight LMICs.

**Methods:**

Data among former and current adult smokers aged 18 and older came from contemporaneous Global Adult Tobacco Surveys (2008–2011) and the International Tobacco Control Surveys (2009–2013) conducted in eight LMICs (Bangladesh, Brazil, China, India, Mexico, Malaysia, Thailand and Uruguay). Adjusted odds ratios (AORs) of successful quitting in the past year by SES indicators (household income/wealth, education, employment status, and rural-urban residence) were estimated using multivariable logistic regression controlling for socio-demographics and average tobacco product prices. A random effects meta-analysis was used to combine the estimates of AORs pooled across countries and two concurrent surveys for each country.

**Results:**

Estimated quit rates among smokers (both daily and occasional) varied widely across countries. Meta-analysis of pooled AORs across countries and data sources indicated that there was no clear evidence of an association between SES indicators and successful quitting. The only exception was employed smokers, who were less likely to quit than their non-employed counterparts, which included students, homemakers, retirees, and the unemployed (pooled AOR≈0.8, p<0.10).

**Conclusion:**

Lack of clear evidence of the impact of lower SES on adult cessation behaviour in LMICs suggests that lower-SES smokers are not less successful in their attempts to quit than their higher-SES counterparts. Specifically, lack of employment, which is indicative of younger age and lower nicotine dependence for students, or lower personal disposable income and lower affordability for the unemployed and the retirees, may be associated with quitting. Raising taxes and prices of tobacco products that lowers affordability of tobacco products might be a key strategy for inducing cessation behaviour among current smokers and reducing overall tobacco consumption. Because low-SES smokers are more sensitive to price increases, tobacco taxation policy can induce disproportionately larger decreases in tobacco consumption among them and help reduce socio-economic disparities in smoking and consequent health outcomes.

## Introduction

Smoking is often more prevalent among those with lower socio-economic status (SES) [1,2] and has been found to be a leading contributor to socio-economic disparities in mortality and health in European countries [3–5] and in the United States [6]. Smoking can create a disproportionately larger health and economic burden on those with lower SES. This is often due to the higher proportion of income they spend on purchasing tobacco products as well as on treating tobacco-induced diseases. The loss of productivity and income caused by tobacco-attributable morbidity and premature mortality is also a major contributor to the economic burden on lower-SES tobacco users [2]. This socio-economic pattern in smoking and its health and economic consequences are particularly visible in high-income countries, with emerging evidence in the context of low- and middle-income countries (LMICs).

Using the World Health Organization World Health Survey of 70 countries from 2002 to 2003, for example, Fleischer and colleagues observed that current smoking prevalence was generally higher among men of lower education, with exceptions to this pattern in several countries in sub-Saharan Africa [7]. Based on data from the Global Adult Tobacco Survey (GATS) in 13 LMICs, Palipudi and colleagues found higher use of tobacco among individuals of lower education and wealth status, with exceptions in Mexico, Turkey and China [8]. A more recent study based on data from the Demographic and Health Surveys in 54 LMICs found evidence of socio-economic inequalities in tobacco use in most countries among men and women [9].

Socio-economic inequalities in smoking can be driven by disparities in both initiation and cessation of smoking across different SES. This paper is focuses on the socio-economic patterns in quitting behaviour of smokers in LMICs. There may be several reasons to expect that smokers with lower SES may be less successful in quitting. They include reduced support for quitting, low motivation to quit, stronger addiction to tobacco, psychological differences, increased likelihood of not finishing courses of pharmacotherapy and behavioral support, and targeted marketing by tobacco companies [10]. In a systematic review, Twyman and colleagues also identified multiple perceived barriers to smoking cessation in vulnerable groups that include—(i) individual and lifestyle factors; (ii) social and community factors; (iii) living conditions; and (iv) cultural, socio-economic and environmental factors [11]. Smoking cessation among low-SES smokers might also be undermined by the need for nicotine to suppress appetite and manage hunger [12, 13].

Existing evidence on differences in quitting smoking by SES of tobacco users along different dimensions—such as, income, wealth, education, occupation, and residence in deprived areas—mostly pertains to high-income countries [14–34]. These studies have consistently found that lower SES is predictive of lower probability of quit intention, quit attempts and successful quitting.

Less, however, is known about whether those with lower SES have varying likelihood of successfully quitting compared to those with higher SES in LMICs. The limited evidence available from LMICs is inconclusive regarding the association between smoking cessation behaviour and SES. International Tobacco Control (ITC) survey data from Bangladesh, Brazil, Malaysia, and Thailand found no association between cessation and education or income [35–37]. However, the ITC Brazil survey did find greater likelihood of cessation attempts among high SES smokers who received physician advice to quit compared to low-SES smokers [36]. Similarly, successful quitting has been found to be associated with higher SES in Hong Kong [38], Vietnam [39], Indonesia [39], Malaysia and Thailand [40], China [41] and India [42]. In the ITC Mexico survey, university education, but not income, was associated with subsequent smoking cessation in the early waves [43]; this association was not found, however, in later waves [44]. Given the mixed findings, more studies would be needed to systematically examine the relationship between indicators of SES and quitting outcomes in LMICs.

To address this existing gap in the literature, this paper examines socio-economic patterns in cessation behaviour of individual smokers in LMICs, where substantial progress has been made in implementing tobacco control policies over the last decades [45]. Specifically, this paper examines whether successful quitting varies by SES using data from eight LMICs at the time of GATS and ITC survey implementation, including Bangladesh, Brazil, China, India, Malaysia, Mexico, Thailand, and Uruguay. The SES of individuals is represented by residence in rural or urban areas, employment status, and education in both GATS and ITC Surveys, in addition to a “wealth index” in GATS and household income group in the ITC Surveys. Although area of residence is not a common indicator of SES, it is strongly correlated with housing conditions that measure material aspects of socio-economic circumstances [46]. Therefore, in this paper, rural residence has been used as a proxy measure of lower SES. The research question in this paper is important to address because if the success rate of quitting is lower or negligible among the lower-SES smokers, the health and economic burden of smoking is likely to increase for them, worsening health inequalities in LMICs.

## Materials and methods

### Data

This research is based on data from the International Tobacco Control (ITC) Surveys and Global Adult Tobacco Surveys (GATS) conducted in Bangladesh (GATS, 2009; ITC, 2009, 2010), Brazil (GATS, 2008; ITC, 2009, 2012/13), China (GATS, 2010; ITC, 2009, 2011/12), India (GATS, 2009/2010; ITC, 2010/2011, 2012/2013), Malaysia (GATS, 2011; ITC, 2009, 2011/2012), Mexico (GATS, 2009; ITC, 2008, 2010), Thailand (GATS, 2011; ITC, 2009, 2011) and Uruguay (GATS, 2009; ITC, 2008/2009, 2010/2011).

The survey protocols and all materials, including the survey questionnaires for all ITC country surveys, were cleared for ethics by Office of Research Ethics, University of Waterloo, Canada. Additional ethics clearances were also obtained from Roswell Park Cancer Institute International Review Board, USA; Cancer Council Victoria International Review Board, Australia; Mahidol University International Review Board, Thailand; National Cancer Institute of Brazil (INCA) International Review Board, Brazil; Bangladesh Medical Research Council, Bangladesh; the Healis Sekhsaria Institute for Public Health International Research Board, India; Universiti Sains Malaysia International Review Board, Malaysia; Chinese Center for Disease Control and Prevention International Review Board, China and the Instituo Nacional de Salud Publica, International Research Board, Mexico.

The use of two different sources of data for the same set of countries allows us to check the validity and consistency of results for each country. S1 Tables (Table A1) lists the survey waves and years (included in the analysis) of ITC and GATS. All the countries were in low, lower-middle or upper-middle income status during the survey years according to the World Bank economic classification [47]. Uruguay moved to high income status in 2012.

#### International Tobacco Control Survey (ITC)

The ITC surveys provide cohort data on the quitting behaviour of adult smokers aged 18 years or older in 28 countries (e.g. Australia, Bangladesh, Bhutan, Brazil, Canada, China, France, Germany, Greece, Hungary, India, Ireland, Kenya, Malaysia, Mauritius, Mexico, Netherlands, New Zealand, Poland, Republic of Korea, Romania, Spain, Thailand, United Arab Emirates, United Kingdom, United States, Uruguay, and Zambia), except in Bangladesh and India where the survey included tobacco users (including both smokers and smokeless tobacco users) aged 15 years or older. For the purpose of comparability in analysis, the samples in Bangladesh and India were restricted to the 18 years or older groups. The sample size ranged from 915 respondents in Uruguay to 3,425 respondents in China; the sample retention rate in two consecutive waves ranged from 64.9% in Mexico to 94.2% in Bangladesh (Table 1). The response rate could not be calculated in a comparable way for all countries included in the study.

**Table 1:** Characteristics of the study samples (current smokers and quitters) by survey and country.

**GATS sample**	**Bangladesh**		**Brazil**		**China**		**India**		**Malaysia**		**Mexico**		**Thailand**		**Uruguay**	
	**%**	**(Freq.)**	**%**	**(Freq.)**	**%**	**(Freq.)**	**%**	**(Freq.)**	**%**	**(Freq.)**	**%**	**(Freq.)**	**%**	**(Freq.)**	**%**	**(Freq.)**
**Gender**	** **	** **														
Men	96.5	(2252)	57.2	(4213)	93.8	(3941)	88.4	(10608)	96.4	(975)	75.8	(1642)	94.3	(4076)	57.2	(900)
Women	3.5	(81)	42.8	(3156)	6.2	(259)	11.6	(1397)	3.6	(36)	24.2	(524)	5.7	(423)	42.8	(673)
**Age Group**																
18–24	10.8	(252)	12.9	(948)	4.5	(191)	8.3	(991)	14.3	(145)	24.8	(538)	17.3	(448)	14.8	(233)
25–34	25.8	(602)	22.2	(1634)	12.3	(515)	21.4	(2565)	26.4	(267)	24.6	(532)	19.4	(745)	23.6	(372)
35–44	28.7	(670)	22.3	(1645)	24.7	(1038)	28.9	(3472)	22.6	(228)	19.9	(430)	24.2	(991)	18.8	(296)
45–54	18.0	(419)	21.3	(1569)	23.7	(995)	20.7	(2491)	19.4	(196)	14.8	(321)	18.7	(1028)	19.1	(301)
55–64	10.4	(242)	12.4	(915)	19.4	(815)	11.5	(1378)	11.1	(112)	8.9	(193)	13.1	(719)	14.0	(221)
65+	6.3	(148)	8.9	(658)	15.4	(646)	9.2	(1108)	6.2	(63)	7.0	(152)	7.4	(568)	9.5	(150)
**Residence**																
Urban	47.6	(1111)	81.9	(6033)	37.9	(1590)	31.7	(3801)	46.1	(466)	63.5	(1376)	29.6	(2405)	66.3	(1043)
Rural	52.4	(1222)	18.1	(1336)	62.1	(2610)	68.3	(8204)	53.9	(545)	36.5	(790)	70.4	(2094)	33.7	(530)
**Employment status**																
Employed	86.4	(2015)	NA		85.7	(3599)	79.1	(9475)	85.6	(865)		(1482)	85.5	(3803)	70.5	(1109)
Not employed	13.6	(318)	NA		14.3	(599)	20.9	(2499)	14.4	(145)	31.3	(675)	14.5	(696)	29.5	(464)
**Education**																
Low	63.9	(1491)	23.9	(1761)	35.3	(1483)	46.1	(5512)	12.5	(126)	20.6	(445)	58.1	(2639)	57.1	(898)
Middle	24.5	(571)	68.3	(5033)	55.9	(2346)	46.3	(5544)	80.0	(809)	72.6	(1570)	34.5	(1390)	36.1	(568)
High	11.6	(271)	7.8	(575)	8.8	(370)	7.6	(911)	7.5	(76)	6.8	(147)	7.5	(469)	6.8	(107)
**Household Wealth**																
Low	39.3	(916)	NA		27.9	(1171)	39.2	(4702)	35.3	(357)	24.8	(538)	37.8	(1852)	37.4	(589)
Middle	34.5	(805)	NA		37.3	(1565)	35.5	(4265)	39.0	(394)	33.3	(721)	33.9	(1428)	32.9	(518)
High	26.2	(612)	NA		34.9	(1464)	25.3	(3038)	25.7	(260)	41.9	(907)	28.3	(1219)	29.6	(466)
**ITC sample**	**Bangladesh**		**Brazil**		**China**		**India**		**Malaysia**		**Mexico**		**Thailand**		**Uruguay**	
** **	**%**	**(Freq.)**	**%**	**(Freq.)**	**%**	**(Freq.)**	**%**	**(Freq.)**	**%**	**(Freq.)**	**%**	**(Freq.)**	**%**	**(Freq.)**	**%**	**(Freq.)**
**Gender**	** **	** **														
Male	96.1	(2086)	42.7	(519)	95.2	(3260)	98.2	(1743)	99.4	(1397)	61.2	(735)	90.3	(1360)	48.5	(444)
Female	3.9	(84)	57.3	(696)	4.8	(165)	1.8	(32)	0.6	(9)	38.8	(466)	9.7	(146)	51.5	(471)
**Age group**	** **	** **														
18–24	16.4	(356)	9.3	(113)	1.4	(48)	9.1	(162)	36.3	(507)	18.2	(219)	5.0	(76)	17.9	(164)
25–34	26.5	(575)	17.4	(211)	9.7	(332)	21.0	(372)	23.7	(331)	23.9	(287)	13.5	(204)	23.2	(212)
35–44	18.1	(393)	21.0	(255)	21.3	(729)	25.1	(446)	17.6	(246)	21.1	(253)	23.6	(356)	20.5	(188)
45–54	17.6	(382)	29.8	(362)	33.4	(1143)	22.8	(404)	14.4	(201)	18.8	(226)	27.6	(416)	20.1	(184)
55–64	12.6	(274)	15.6	(190)	19.7	(674)	14.4	(256)	8.0	(112)	10.8	(130)	17.7	(267)	12.9	(118)
65+	8.8	(190)	6.9	(84)	14.6	(499)	7.6	(135)			7.2	(86)	12.4	(187)	5.4	(49)
**Marital status**	** **	** **														
Married	80.5	(1742)	47.3	(574)	88.7	(3031)	82.0	(1456)	NA		56.5	(678)	NA		41.2	(377)
Otherwise	19.5	(421)	52.7	(639)	11.3	(385)	18.0	(319)	NA		43.5	(522)	NA		58.8	(538)
**Residence**	** **	** **														
Urban	34.4	(746)	100.0	(1215)	100.0	(3425)	70.8	(1257)	62.1	(873)	100.0	(1201)	41.7	(628)	100.0	(915)
Rural	65.6	(1424)	NA		NA		29.2	(518)	37.9	(533)	NA		58.3	(878)	NA	
**Employment status**	** **	** **														
Employed	45.1	(975)	63.4	(770)	60.7	(2057)	77.6	(1382)	79.5	(1116)	63.1	(757)	77.4	(1164)	64.4	(588)
Not employed	54.9	(1189)	36.6	(445)	39.3	(1331)	22.4	(400)	20.5	(287)	36.9	(442)	22.6	(339)	35.6	(325)
**Education**	** **	** **														
Low	23.0	(499)	33.4	(405)	11.7	(400)	58.1	(1028)	8.7	(120)	29.7	(352)	71.7	(1075)	61.1	(558)
Middle	53.2	(1152)	38.5	(466)	64.2	(2194)	26.7	(472)	81.9	(1124)	55.3	(655)	20.5	(307)	22.9	(209)
High	23.7	(514)	28.1	(340)	24.1	(823)	15.2	(268)	9.3	(128)	15.0	(178)	7.9	(118)	16.1	(147)
**Household Income**	** **	** **														
Low	16.0	(348)	38.7	(470)	10.1	(347)	33.6	(597)	27.7	(389)	25.2	(303)	32.1	(483)	37.9	(346)
Middle	42.7	(927)	40.3	(490)	38.0	(1299)	50.0	(887)	29.1	(409)	24.7	(297)	34.1	(513)	46.9	(428)
High	29.4	(638)	13.9	(169)	46.8	(1600)	14.0	(248)	27.0	(379)	37.1	(446)	32.1	(484)	8.8	(80)

Note: The percentages for household income categories in ITC sample do not add up to 100% because the “not stated” category is not reported for the sake of simplicity.

#### Global Adult Tobacco Survey (GATS)

GATS is a nationally representative household survey completed in 28 countries (e.g. Argentina, Bangladesh, Brazil, Cameroon, China, Egypt, Greece, India, Indonesia, Kazakhstan, Kenya, Malaysia, Mexico, Nigeria, Panama, Pakistan, Philippines, Poland, Qatar, Romania, Russian Federation, Senegal, Thailand, Turkey, Uganda, Ukraine, Uruguay, Viet Nam), providing comprehensive data on current and former tobacco use and other key tobacco control indicators including data on SES among adults aged 15 and older. For this analysis, only adults aged 18 years or older were included.

The survey is cross-sectional and uses a global standardized protocol aimed at enhancing countries’ capacity to monitor tobacco use, facilitating tobacco data analysis at the country and regional levels, and guiding the tobacco control and prevention programs of the countries. The GATS data are available for one year only for each country except for Thailand, where two waves of the survey were conducted in 2009 and 2011. These are, however, cross-sectional surveys based on independent observations in the two waves. For the present study, data were used from the latest wave only which was conducted in 2011 and coincided with an ITC survey year. The sample size in GATS ranged from 1,011 respondents in Malaysia to 12,005 respondents in India; the response rate ranged from 82.5% in Mexico to 96.3% in Bangladesh and Thailand (Table 1). Further details of the GATS can be found in Palipudi et al. (2016) [48].

### Outcome measure

The outcome of interest is the quit rate defined as the ratio of the number of self-reported quitters to the total number of current and former smokers, both daily and occasional. The measures for quitting vary across different studies as the process of smoking cessation involves at least three important stages: intending to quit, attempting to quit, and succeeding in quitting. Based on recommendations of the U.S. Preventive Services Task Force (USPSTF), the present study uses abstinence from smoking for at least 6 months within the past one year to define successful quitting as the outcome measure of interest [49].

In GATS, current tobacco smokers were identified by responses to the question, “Do you currently smoke tobacco on a daily basis, less than daily, or not at all?” Those who responded that they smoked tobacco "daily" or "less than daily " (occasional) were classified as current tobacco smokers. Those who responded that they did not currently smoke tobacco were asked, “In the past, have you smoked tobacco on a daily basis, less than daily, or not at all?”. Based on these two questions, former smokers were defined as those who smoked daily or occasionally in the past but not currently.

In the ITC surveys, current smokers were defined as those who responded “yes” to the questions “Have you smoked 100 or more cigarettes over your lifetime?” and “Do you currently smoke, either daily or less than daily?” This question included bidi (hand-rolled traditional smoked tobacco product) in Bangladesh and India, and roll-your-own (RYO) cigarettes in Thailand. Thus, in these three countries, quit rates were estimated among all who smoked daily or occasionally (including users of single and multiple smoked tobacco products).

The difference between GATS and ITC surveys in identifying smoking status of individuals is that ITC looks at cigarette smokers only and screens out those who have smoked less than 100 cigarettes over their lifetime, whereas GATS looks at tobacco smokers (any combustible tobacco product) and does not screen out by quantity used. In the absence of information on lifetime cigarette consumption in the GATS data, we were not able to make the identification of smoking status in the two surveys identical.

In all countries, ITC respondents who were smokers at Wave t were followed up at Wave t+1 to estimate quit rates, defined as the proportion of smokers at Wave t who reported no longer smoking at Wave t+1. In contrast, GATS is a cross-sectional survey and we were not able to determine whether the smokers quit in subsequent waves. To make GATS comparable to the ITC survey, the quit rate was defined as the proportion of past year smokers (i.e., total number of current smokers and former smokers who quit in the last 12 months) who no longer smoked tobacco.

In GATS, former smokers were asked, “How long has it been since you stopped smoking?”, whereas in ITC, former smokers were asked, “How long ago did you quit?”. To check the robustness of results, we used different durations of abstinence to define successful quitting: currently quit for any duration, at least 1 month, at least 3 months, and at least 6 months in last 12 months. Quit for one year or longer was not included because of lack of observations required for the adjusted analysis.

We also used a measure of whether a smoker made any quit attempts in the past one year. In GATS, current smokers were asked, “During the past 12 months have you tried to stop smoking?”. Those who replied “yes” to this question and all former smokers were coded as “have made a quit attempt”. In ITC, current smokers were asked, “Since we last talked to you, how many times have you tried to quit smoking?”. All who had tried to quit at least once were followed up with “Since we last talked to you, how long ago did your most recent quit attempt fail?” Those who answered that it was less than 12 months and all former smokers were coded as “have made a quit attempt”.

In Bangladesh and India, where smokeless tobacco is widely used, some tobacco smokers who quit smoking may transition to or continue to use smokeless tobacco. In these cases, smokers who quit were classified as having quit smoking tobacco but not having quit smokeless tobacco. In a separate analysis, smokers who quit smoking and did not use smokeless tobacco were classified as quitting completely for both GATS and ITC surveys.

### Predictor variables

#### SES indicators

In both surveys, respondents were classified by whether they came from urban or rural areas. In the ITC surveys in China, Mexico and Uruguay, however, only smokers from urban areas were sampled, so quit rates in these areas could not be estimated by urban/rural residence.

In both surveys, educational status was assessed using the question "What is your highest level of education?" Due to differences in educational systems between countries, only a relative measure of educational attainment could be used. Specifically, respondents were classified into three categories: "low", "middle" and "high". The specific groupings varied by country as shown in S1 Tables (Table A2).

Employment status in the ITC survey was assessed using a single question, "Are you currently employed outside the home?" Smokers answering "yes" were classified as “employed” while those answering "no" were classified as "not employed". In the GATS, individuals reporting themselves as “government employee”, “non-government employee” or “self-employed” were classified as employed while those reporting themselves as “student”, “homemaker”, retired”, “unemployed, able to work”, “unemployed, unable to work” were classified as “not employed”.

For all ITC countries, respondents were classified into three income groups (low/middle/high) based on either annual or monthly household income reported in a specific income range as a categorical variable. The classification scheme was based on information provided by the local ITC team in each country as to which cut points constituted low, middle, or high-income groups or the approximate tertiles. Malaysia and Thailand were exceptions where respondents reported household income as a continuous measure. For these two countries, a per capita measure of household income was constructed and then ranked into approximate tertiles to construct the low, middle, and high-income categories. The specific groupings of income by country are shown in S1 Tables (Table A3). These income measures were created as a standard way to classify income across all ITC countries and have been used successfully in past analyses and publications. The purpose of creating a relative measure of income was to be able to compare relative income effects across countries without having to convert an absolute measure of income to a common international currency or to the poverty status of respondents. In the absence of a continuous measure of income for the other countries, it is not feasible to use more granular income classifications (e.g., quintiles or deciles).

Household income data are not available in GATS. Studies using GATS data generally construct a wealth index (based on household ownership of assets and access to utility services) as a proxy measure for respondent SES and the sample is then divided into quintiles based on this wealth index [8, 50]. For the present analysis, the same wealth index was used to divide the sample into wealth tertiles described as “low”, “middle” and “high” SES comparable to ITC income tertiles for the same country.

All predictor variables used in the ITC data were measured at Wave t, while all quit rates were measured at Wave t+1. Where appropriate, adjusted estimates controlled for time-in-sample, i.e., the number of times a respondent had participated in the ITC survey (this is only applicable when Wave t > 1, e.g., where the initial wave for the analysis is Wave 2 or higher). It is important to adjust for time-in-sample effect as ITC surveys replenish respondents lost to attrition in all waves after the first one.

#### Socio-demographics

The socio-demographic and socio-economic characteristics of the study samples are summarized in Table 1 by country and survey. Respondents’ socio-demographic characteristics that were assessed included: gender, age and marital status (marital status available only in ITC data). The age variable was based on self-reported age grouped into six categories: 18–24, 25–34, 35–44, 45–54, 55–64 and 65+ years. Marital status in ITC was classified as either "married" or "otherwise", which covered “divorced or separated", "widowed" or "single". In addition, city/state (available only in ITC data) was included as a predictor.

Assessments of quit rates controlled for local cigarette prices in price per stick, which were based on purchasing prices for cigarettes reported by current smokers and averaged to the "village" level or smallest level of geographical unit available in the respective survey for that country. The use of geographical average price serves two purposes. First, it represents the general price level faced by all respondents, including current and former smokers (self-reported prices are not available for former smokers), at the time of survey in the corresponding region, and influences their smoking decision. Second, using geographical average price as an instrument for price variable helps address the endogeneity bias caused by self-reported price which is determined simultaneously with smoking behavior. Because a sizeable number of tobacco smokers in India and Bangladesh smoked bidis, local tobacco prices were based on average reported prices for both cigarettes and for bidis. In Thailand, a large percentage of smokers smoke RYO cigarettes, so quit rates controlled for average cigarette price for smokers of factory-made cigarettes and average pouch price for smokers of RYO cigarettes.

## Statistical analysis

For each country, the following multivariate logistic regression was used to examine the association between SES variables and quitting outcomes adjusting for socio-demographic variables, such as, age, gender and marital status:

$$P(Q_{i}|D,X)=\frac{exp(\beta_{0}+{SES}_{i}^{\prime }\gamma_{t}+x_{i}^{\prime }\beta_{j})}{1+exp(\beta_{0}+{SES}_{i}^{\prime }\gamma_{t}+x_{i}^{\prime }\beta_{j})}$$

where *Q_i_* takes the value of 1 if individual *i* reported abstinence from smoking for at least 6 months within the past one year, and 0 otherwise. *SES* is a vector of dummies for the SES indicators, such as, residence (urban, rural), employment status (employed, not employed), education (low, middle, high), and household income/wealth (low, middle, high). *X* is a vector of dummies for the socio-demographic covariates (e.g., gender, age and marital status), dummies for the type of smoked tobacco product (e.g., cigarette, bidi, RYO, and dual use) used by the smoker in the past, and corresponding tobacco product prices.

Adjusted odds ratio (AOR) of quit rates with 95% confidence intervals for each comparison of SES indicators (e.g. high vs. low income within the income categories) was estimated. The regression analysis was conducted separately for each country accounting for complex, multi-stage survey design applied to each country. We generated results from 80 regressions for five cessation measures for eight countries using two datasets. For each regression, we tested the significance of the marginal effects of different categories of each SES indicator using individual t-tests as well as the significance of overall effects using Wald chi-square tests. Detailed results of estimation are available in S2–S5 Tables. The statistical packages used for data analysis include SAS 9.4, SAS-callable SUDAAN 11.0.1 and Stata 14.0.

All estimates in ITC data analysis were weighted using the longitudinal sampling weight for respondents present in both Wave t and Wave t+1, except for Brazil. Due to the long interval between Waves 1 and 2 in Brazil, the retention rate in Wave 2 was low. In this case, quit rates were estimated using an "intention-to-treat" approach, where smokers lost to attrition were assumed to be smokers in Wave 2 (which may result in a conservative estimate of quit rate). Thus, the cross-sectional sampling weight from Wave 1 was used to estimate quit rates in Wave 2 for the Brazilian sample. For GATS data, all estimates were weighted by cross-sectional individual sampling weights.

A random-effects meta-analysis of country AORs (effect size) was conducted for pairs of values of each SES indicator to combine the country-level results and identify any systematic pattern in the odds to quit by SES across all countries. This approach allows the effect size to vary from country to country, in contrast to a fixed effect model which assumes that the effect size is the same in all countries. The estimate from each survey for each country was treated as a separate estimate. In the first stage, the estimates for the eight countries were combined into a summary measure (called pooled AOR) for each survey. Each estimate was weighted by the inverse of its variance. In the second stage, two pooled AORs for GATS and ITC surveys were combined into a single AOR, again weighting by the inverse of the variance of each AOR pooled by surveys. Based on prior information from studies done in high-income countries, we hypothesized that the pooled AOR>1, which would suggest that higher SES is predictive of higher odds of quitting. The heterogeneity of AORs across surveys was tested for each country using the I-squared test statistic that represents the percentage of observed variance between studies that is due to differences in the effect size. The statistical significance of pooled AORs was tested using a p-value of 0.10, rather than the conventional level of 0.05, following the general methods for identifying and measuring heterogeneity in Cochrane reviews [51].

## Results

Overall quit rates by different cessation measures varied widely across countries (Table 2). The percentage of smokers (both daily and occasional) who quit smoking for at least 6 months in the last 12 months ranged from 1.6% in Malaysia to 8.7% in Mexico in GATS, and from 1.7% in Brazil and India to 8.8% in Mexico in ITC. The quit rates are higher for measures less restrictive on the duration of abstinence. The percentage of smokers (both daily and occasional) who quit smoking for at least 3 months in the last 12 months varied from 1.9% in Malaysia to 12.6% in Mexico in GATS, and from 2.4% in India to 11.2% in Mexico in ITC. The percentage of smokers (both daily and occasional) who quit smoking for at least 1 month in last 12 months varied from 2.7% in Malaysia to 15.2% in Mexico in GATS, and from 2.8% in India to 14.3% in Mexico in ITC. Quit rates in the last 12 months, without restriction on the duration of abstinence, were between 3.0% in Malaysia and 16.0% in Mexico in GATS, and between 4.8% in Brazil and 14.7% in Mexico in ITC. In Bangladesh and India, the percentage of smokers and smokeless tobacco users (both daily and occasional) who quit tobacco use altogether was 2.6% in India and 3.1% in Bangladesh according to GATS, and 3.6% in India and 4.6% in Bangladesh according to ITC. The rate of quit attempts in the last 12 months varied from 14.4% in China to 49.9% in Mexico according to GATS, and from 21.0% in India to 73.9% in Thailand according to ITC.

**Table 2:** Overall quit rates and adjusted odds ratios (AOR) of quit rates by different indicators of socio-economic status, duration of abstinence, countries and surveys.

**BANGLADESH**												
**GATS**	**Quit Smoking in last 12 months**		**Quit Smoking for at least 1 month in last 12 months**		**Quit Smoking for at least 3 months in last 12 months**		**Quit Smoking for at least 6 months in last 12 months**		**Quit Tobacco (smoking and smokeless) in last 12 months**		**Quit Attempts in last 12 months**	
**Overall quit rate (%)**	5.1	(3.7–6.8)	4.3	(3.3–5.7)	3.5	(2.6–4.7)	2.6	(1.8–3.6)	3.1	(2.0–4.9)	47.3	(43.9–50.8)
**Adjusted odds ratio**												
**Household Wealth **												
Middle vs Low	0.89	(0.41–1.94)	0.90	(0.40–1.99)	0.68	(0.31–1.51)	0.80	(0.31–2.05)	1.01	(0.34–2.99)	1.11	(0.84–1.47)
High vs Low	2.18	(0.77–6.18)	1.18	(0.56–2.51)	1.10	(0.49–2.44)	1.21	(0.45–3.26)	3.07	(0.71–13.28)	1.52	(1.07–2.14)
**Education**												
Middle vs Low	0.42	(0.17–1.05)	0.58	(0.28–1.19)	0.71	(0.36–1.42)	0.69	(0.30–1.57)	0.44	(0.13–1.52)	1.09	(0.81–1.45)
High vs Low	0.57	(0.16–2.04)	1.13	(0.39–3.27)	1.45	(0.50–4.20)	1.61	(0.43–6.04)	0.58	(0.11–2.98)	1.05	(0.65–1.68)
**Residence**												
Urban vs Rural	1.38	(0.76–2.50)	0.99	(0.56–1.76)	1.14	(0.63–2.07)	0.95	(0.50–1.78)	1.99	(0.91–4.35)	1.24	(0.94–1.62)
**Employment Status**												
Employed vs Not Employed	0.67	(0.33–1.38)	0.61	(0.28–1.32)	0.61	(0.26–1.41)	0.57	(0.22–1.47)	0.61	(0.28–1.33)	0.94	(0.65–1.36)
**ITC**	**Quit Smoking in last 12 months**		**Quit Smoking for at least 1 month in last 12 months**		**Quit Smoking for at least 3 months in last 12 months**		**Quit Smoking for at least 6 months in last 12 months**		**Quit Tobacco (smoking and smokeless) in last 12 months**		**Quit Attempts in last 12 months**	
**Overall quit rate (%)**	7.0	(5.2–9.1)	4.2	(3.2–5.5)	3.6	(2.7–4.7)	2.3	(1.5–3.3)	4.6	(3.5–6.0)	25.0	(21.0–29.2)
**Adjusted odds ratio**												
**Household Income**												
Middle vs Low	0.95	(0.50–1.81)	1.17	(0.57–2.40)	0.88	(0.41–1.91)	0.55	(0.23–1.33)	1.18	(0.59–2.37)	1.02	(0.62–1.68)
High vs Low	1.03	(0.53–2.00)	0.88	(0.45–1.71)	0.86	(0.42–1.78)	0.68	(0.29–1.58)	1.00	(0.49–2.04)	1.53	(1.17–2.02)
**Education**												
Middle vs Low	0.70	(0.39–1.24)	0.69	(0.30–1.62)	1.17	(0.52–2.67)	1.74	(0.56–5.42)	0.70	(0.32–1.54)	1.02	(0.72–1.45)
High vs Low	0.96	(0.47–1.97)	1.16	(0.50–2.71)	1.88	(0.78–4.55)	2.18	(0.62–7.67)	1.11	(0.50–2.46)	1.39	(1.00–1.94)
**Residence**												
Urban vs Rural	0.59	(0.28–1.23)	0.72	(0.33–1.56)	0.83	(0.38–1.85)	1.03	(0.31–3.35)	0.64	(0.29–1.41)	0.41	(0.25–0.67)
**Employment Status**												
Employed vs Not Employed	0.79	(0.53–1.17)	0.73	(0.45–1.20)	0.49	(0.26–0.91)	0.49	(0.19–1.25)	0.77	(0.48–1.21)	1.48	(1.10–2.01)
**BRAZIL**												
**GATS**	**Quit Smoking in last 12 months**		**Quit Smoking for at least 1 month in last 12 months**		**Quit Smoking for at least 3 months in last 12 months**		**Quit Smoking for at least 6 months in last 12 months**		**Quit Attempts in last 12 months**			
**Overall quit rate (%)**	11.5	(10.7–12.5)	11.0	(10.2–12.0)	9.3	(8.6–10.2)	7.0	(6.3–7.7)	45.6	(44.3–47.0)		
**Adjusted odds ratio**												
**Household Wealth **												
Middle vs Low	NA		NA		NA		NA		NA			
High vs Low	NA		NA		NA		NA		NA			
**Education**												
Middle vs Low	1.22	(0.91–1.65)	1.23	(0.91–1.67)	1.27	(0.92–1.77)	1.17	(0.80–1.70)	0.92	(0.77–1.10)		
High	1.64	(1.08–2.49)	1.70	(1.11–2.59)	1.54	(0.99–2.40)	1.45	(0.87–2.42)	0.78	(0.60–1.02)		
**Residence**												
Urban	0.89	(0.67–1.19)	0.88	(0.65–1.19)	0.79	(0.58–1.07)	0.91	(0.63–1.30)	1.04	(0.87–1.23)		
**Employment Status**												
Employed vs Not Employed	NA		NA		NA		NA		NA			
**ITC**	**Quit Smoking in last 12 months**		**Quit Smoking for at least 1 month in last 12 months**		**Quit Smoking for at least 3 months in last 12 months**		**Quit Smoking for at least 6 months in last 12 months**		**Quit Attempts in last 12 months**			
**Overall quit rate (%)**	4.8	(3.5–6.6)	4.5	(3.2–6.1)	3.4	(2.3–4.9)	1.7	(1.0–2.6)	22.6	(19.7–25.7)		
**Adjusted odds ratio**												
**Household Income**												
Middle vs Low	0.93	(0.39–2.19)	0.81	(0.33–2.01)	1.51	(0.60–3.84)	1.98	(0.60–6.51)	1.48	(0.95–2.30)		
High vs Low	1.43	(0.42–4.88)	1.67	(0.47–5.89)	2.41	(0.55–10.68)	0.71	(0.10–5.08)	1.63	(0.88–3.02)		
**Education**												
Middle vs Low	1.17	(0.47–2.88)	1.13	(0.44–2.91)	0.82	(0.33–2.05)	1.12	(0.30–4.13)	1.38	(0.89–2.12)		
High vs Low	1.13	(0.40–3.17)	0.92	(0.30–2.81)	0.49	(0.13–1.85)	0.71	(0.17–3.00)	1.22	(0.74–2.03)		
**Residence**												
Urban vs Rural	NA		NA		NA		NA		NA			
**Employment Status**												
Employed vs Not Employed	0.97	(0.46–2.06)	0.83	(0.38–1.79)	0.77	(0.33–1.79)	0.83	(0.33–2.12)	1.26	(0.83–1.91)		
**CHINA**												
**GATS**	**Quit Smoking in last 12 months**		**Quit Smoking for at least 1 month in last 12 months**		**Quit Smoking for at least 3 months in last 12 months**		**Quit Smoking for at least 6 months in last 12 months**		**Quit Attempts in last 12 months**			
**Overall quit rate (%)**	4.7	(3.6–6.1)	4.5	(3.4–5.9)	3.3	(2.6–4.2)	2.7	(2.0–3.5)	14.4	(11.9–17.2)		
**Adjusted odds ratio**												
**Household Wealth **												
Middle vs Low	1.29	(0.75–2.21)	1.29	(0.75–2.20)	1.34	(0.73–2.45)	1.15	(0.62–2.12)	0.83	(0.52–1.31)		
High vs Low	1.38	(0.61–3.14)	1.40	(0.60–3.27)	1.31	(0.56–3.05)	1.29	(0.45–3.71)	1.07	(0.58–1.96)		
**Education**												
Middle vs Low	0.58	(0.35–0.97)	0.54	(0.26–1.12)	0.48	(0.24–0.97)	0.33	(0.15–0.70)	1.15	(0.82–1.59)		
High vs Low	1.08	(0.43–2.74)	1.01	(0.40–2.54)	1.15	(0.39–3.33)	1.09	(0.36–3.31)	1.38	(0.83–2.29)		
**Residence**												
Urban vs Rural	0.52	(0.26–1.04)	0.54	(0.26–1.12)	0.64	(0.37–1.11)	0.97	(0.47–2.01)	0.71	(0.46–1.10)		
**Employment Status**												
Employed vs Not Employed	0.24	(0.13–0.45)	0.26	(0.13–0.49)	0.36	(0.17–0.75)	0.31	(0.14–0.69)	0.64	(0.39–1.07)		
**ITC**	**Quit Smoking in last 12 months**		**Quit Smoking for at least 1 month in last 12 months**		**Quit Smoking for at least 3 months in last 12 months**		**Quit Smoking for at least 6 months in last 12 months**		**Quit Attempts in last 12 months**			
**Overall quit rate (%)**	5.8	(4.4–7.5)	5.8	(4.4–7.5)	5.3	(4.0–6.8)	4.2	(3.3–5.4)	22.2	(19.3–25.4)		
**Adjusted odds ratio**												
**Household Income**												
Middle vs Low	0.85	(0.40–1.79)	0.85	(0.40–1.79)	0.66	(0.30–1.43)	1.31	(0.57–3.00)	0.63	(0.38–1.03)		
High vs Low	1.08	(0.47–2.46)	1.08	(0.47–2.46)	0.96	(0.41–2.24)	2.12	(0.87–5.16)	0.54	(0.54–1.45)		
**Education**												
Middle vs Low	0.79	(0.44–1.44)	0.79	(0.44–1.44)	0.74	(0.40–1.39)	0.59	(0.26–1.39)	0.87	(0.60–1.25)		
High vs Low	1.15	(0.61–2.14)	1.15	(0.61–2.14)	1.19	(0.62–2.29)	1.13	(0.50–2.54)	0.88	(0.57–1.36)		
**Residence**												
Urban vs Rural	NA		NA		NA		NA		NA			
**Employment Status**												
Employed vs Not Employed	0.64	(0.38–1.09)	0.64	(0.38–1.09)	0.63	(0.36–1.10)	0.57	(0.30–1.10)	0.90	(0.71–1.15)		
**INDIA**												
**GATS**	**Quit Smoking in last 12 months**		**Quit Smoking for at least 1 month in last 12 months**		**Quit Smoking for at least 3 months in last 12 months**		**Quit Smoking for at least 6 months in last 12 months**		**Quit Tobacco (smoking and smokeless) in last 12 months**		**Quit Attempts in last 12 months**	
**Overall quit rate (%)**	4.6	(3.9–5.3)	3.9	(3.3–4.6)	3.0	(2.5–3.6)	2.2	(1.8 -.2.8)	2.6	(2.1–3.1)	36.4	(34.3–38.5)
**Adjusted odds ratio**												
**Household Wealth **												
Middle vs Low	1.32	(0.90–1.92)	1.43	(0.95–2.15)	1.70	(1.11–2.62)	2.06	(1.26–3.38)	1.69	(1.08–2.64)	1.09	(0.91–1.30)
High vs Low	0.62	(0.39–0.99)	0.63	(0.39–1.04)	0.76	(0.46–1.27)	0.91	(0.51–1.63)	0.87	(0.48–1.55)	1.09	(0.86–1.39)
**Education**												
Middle vs Low	1.47	(1.06–2.04)	1.35	(0.95–1.91)	1.46	(0.98–2.18)	1.46	(0.92–2.33)	1.48	(0.96–2.30)	1.17	(0.98–1.39)
High vs Low	2.62	(1.31–5.23)	2.69	(1.29–5.62)	2.50	(1.04–6.01)	2.57	(0.91–7.27)	3.00	(1.44–6.23)	1.23	(0.87–1.73)
**Residence**												
Urban vs Rural	0.94	(0.65–1.34)	0.93	(0.63–1.39)	0.91	(0.59–1.39)	1.04	(0.64–1.71)	0.94	(0.62–1.43)	0.93	(0.77–1.13)
**Employment Status**												
Employed vs Not Employed	1.10	(0.69–1.76)	1.13	(0.68–1.88)	0.95	(0.55–1.64)	0.85	(0.50–1.45)	1.10	(0.57–2.10)	0.93	(0.73–1.17)
**ITC**	**Quit Smoking in last 12 months**		**Quit Smoking for at least 1 month in last 12 months**		**Quit Smoking for at least 3 months in last 12 months**		**Quit Smoking for at least 6 months in last 12 months**		**Quit Tobacco (smoking and smokeless) in last 12 months**		**Quit Attempts in last 12 months**	
**Overall quit rate (%)**	7.3	(5.2–9.9)	2.8	(1.9–4.0)	2.4	(1.6–3.5)	1.7	(1.1–2.6)	3.6	(2.4–5.0)	21.0	(16.7–25.7)
**Adjusted odds ratio**												
**Household Income**												
Middle vs Low	1.62	(0.92–2.86)	0.76	(0.27–2.16)	0.74	(0.26–2.09)	0.56	(0.16–1.92)	1.19	(0.36–3.95)	1.53	(1.07–2.19)
High vs Low	2.26	(0.87–5.86)	2.10	(0.48–9.26)	1.98	(0.41–9.61)	2.17	(0.44–10.65)	2.90	(0.74–11.42)	1.74	(1.02–2.96)
**Education**												
Middle vs Low	0.82	(0.47–1.43)	1.11	(0.46–2.67)	1.03	(0.42–2.55)	0.69	(0.23–2.01)	1.08	(0.49–2.36)	0.83	(0.54–1.26)
High vs Low	0.29	(0.12–0.68)	0.51	(0.14–1.93)	0.62	(0.19–2.06)	0.59	(0.18–1.93)	0.49	(0.16–1.51)	0.78	(0.53–1.17)
**Residence**												
Urban vs Rural	0.94	(0.40–2.23)	1.04	(0.43–2.54)	1.37	(0.54–3.46)	1.53	(0.45–5.17)	1.06	(0.40–2.77)	0.74	(0.41–1.31)
**Employment Status**												
Employed vs Not Employed	0.74	(0.37–1.47)	0.49	(0.16–1.47)	0.61	(0.24–1.58)	1.03	(0.32–3.32)	0.46	(0.16–1.28)	0.96	(0.61–1.50)
**MALAYSIA**												
**GATS**	**Quit Smoking in last 12 months**		**Quit Smoking for at least 1 month in last 12 months**		**Quit Smoking for at least 3 months in last 12 months**		**Quit Smoking for at least 6 months in last 12 months**		**Quit Attempts in last 12 months**			
**Overall quit rate (%)**	3.0	(1.5–6.0)	2.7	(1.3–5.7)	1.9	(0.9–3.8)	1.6	(0.7–3.5)	48.6	(44.1–53.2)		
**Adjusted odds ratio**												
**Household Wealth **												
Middle vs Low	0.67	(0.20–2.27)	0.48	(0.13–1.83)	1.35	(0.33–5.54)	1.85	(0.37–9.24)	1.30	(0.85–1.99)		
High vs Low	1.54	(0.27–8.86)	1.65	(0.29–9.43)	3.75	(0.81–17.43)	4.16	(0.66–26.12)	0.99	(0.60–1.64)		
**Education**												
Middle vs Low	0.52	(0.10–2.58)	0.50	(0.10–2.39)	2.08	(0.32–13.40)	1.10	(0.08–15.37)	1.20	(0.67–2.14)		
High vs Low	0.78	(0.05–11.62)	0.86	(0.06–12.58)	2.22	(0.14–35.21)	1.28	(0.04–39.18)	1.72	(0.76–3.89)		
**Residence**												
Urban vs Rural	2.93	(1.00–8.63)	2.44	(0.81–7.40)	1.49	(0.48–4.64)	1.43	(0.39–5.29)	1.36	(0.95–1.95)		
**Employment Status**												
Employed vs Not Employed	0.39	(0.10–1.52)	0.56	(0.13–2.39)	0.39	(0.07–2.23)	0.29	(0.04–2.34)	0.65	(0.35–1.18)		
**ITC**	**Quit Smoking in last 12 months**		**Quit Smoking for at least 1 month in last 12 months**		**Quit Smoking for at least 3 months in last 12 months**		**Quit Smoking for at least 6 months in last 12 months**		**Quit Attempts in last 12 months**			
**Overall quit rate (%)**	6.4	(4.9–8.1)	5.9	(4.5–7.7)	4.7	(3.5–6.2)	2.2	(1.4–3.3)	45.1	(38.4–51.9)		
**Adjusted odds ratio**												
**Household Income**												
Middle vs Low	2.02	(0.81–5.04)	1.76	(0.69–4.52)	1.73	(0.58–5.17)	1.09	(0.23–5.09)	1.03	(0.60–1.78)		
High vs Low	1.29	(0.81–2.04)	1.15	(0.74–1.77)	1.40	(0.61–3.22)	1.67	(0.69–4.04)	1.08	(0.66–1.76)		
**Education**												
Middle vs Low	1.07	(0.51–2.26)	1.36	(0.57–3.28)	2.63	(0.42–16.40)	1.16	(0.19–7.04)	0.87	(0.49–1.53)		
High vs Low	0.63	(0.14–2.86)	0.86	(0.19–3.92)	1.52	(0.13–17.63)	1.46	(0.12–17.24)	0.86	(0.34–2.19)		
**Residence**												
Urban vs Rural	1.75	(1.04–2.95)	1.90	(1.12–3.23)	2.63	(1.46–4.76)	1.77	(0.67–4.70)	1.18	(0.80–1.74)		
**Employment Status**												
Employed vs Not Employed	0.98	(0.53–1.80)	1.00	(0.54–1.86)	1.17	(0.71–1.93)	1.69	(0.74–3.90)	0.88	(0.56–1.38)		
**MEXICO**												
**GATS**	**Quit Smoking in last 12 months**		**Quit Smoking for at least 1 month in last 12 months**		**Quit Smoking for at least 3 months in last 12 months**		**Quit Smoking for at least 6 months in last 12 months**		**Quit Attempts in last 12 months**			
**Overall quit rate (%)**	16.0	(14.1–18.2)	15.2	(13.2–17.4)	12.6	(10.8–14.7)	8.7	(6.9–10.8)	49.9	(46.9–53.0)		
**Adjusted odds ratio**												
**Household Wealth **												
Middle vs Low	0.79	(0.51–1.23)	0.78	(0.50–1.22)	0.84	(0.52–1.35)	1.15	(0.62–2.16)	0.94	(0.67–1.32)		
High vs Low	0.67	(0.42–1.08)	0.65	(0.40–1.06)	0.62	(0.35–1.07)	0.95	(0.52–1.74)	0.86	(0.61–1.22)		
**Education**												
Middle vs Low	1.18	(0.72–1.92)	1.30	(0.79–2.14)	1.21	(0.72–2.05)	1.09	(0.56–2.11)	0.93	(0.65–1.35)		
High vs Low	1.10	(0.49–2.49)	1.28	(0.57–2.91)	1.32	(0.53–3.27)	1.12	(0.38–3.33)	0.72	(0.42–1.25)		
**Residence**												
Urban vs Rural	0.73	(0.52–1.04)	0.78	(0.54–1.12)	0.91	(0.62–1.33)	0.79	(0.51–1.23)	0.73	(0.58–0.93)		
**Employment Status**												
Employed vs Not Employed	0.89	(0.62–1.27)	0.86	(0.60–1.24)	0.79	(0.52–1.19)	0.78	(0.46–1.33)	0.85	(0.67–1.08)		
**ITC**	**Quit Smoking in last 12 months**		**Quit Smoking for at least 1 month in last 12 months**		**Quit Smoking for at least 3 months in last 12 months**		**Quit Smoking for at least 6 months in last 12 months**		**Quit Attempts in last 12 months**			
**Overall quit rate (%)**	14.7	(11.3–18.6)	14.3	(10.9–18.3)	11.2	(8.4–14.6)	8.8	(6.3–11.9)	41.8	(36.3–47.4)			
**Adjusted odds ratio**												
**Household Income**												
Middle vs Low	1.02	(0.48–2.15)	1.04	(0.48–2.26)	1.31	(0.41–4.15)	1.38	(0.44–4.36)	0.79	(0.46–1.35)		
High vs Low	1.10	(0.53–2.27)	1.18	(0.58–2.41)	1.70	(0.67–4.27)	2.33	(0.80–6.80)	0.63	(0.38–1.05)		
**Education**												
Middle vs Low	0.90	(0.46–1.75)	0.84	(0.43–1.65)	0.68	(0.30–1.52)	0.46	(0.18–1.19)	0.97	(0.60–1.56)		
High vs Low	2.01	(0.73–5.53)	1.90	(0.70–5.18)	1.04	(0.33–3.28)	0.70	(0.20–2.42)	1.10	(0.54–2.24)		
**Residence**												
Urban vs Rural	NA		NA		NA		NA		NA			
**Employment Status**												
Employed vs Not Employed	1.59	(0.82–3.08)	1.73	(0.90–3.34)	1.31	(0.65–2.67)	1.67	(0.72–3.89)	1.20	(0.77–1.89)		
**THAILAND**												
**GATS**	**Quit Smoking in last 12 months**		**Quit Smoking for at least 1 month in last 12 months**		**Quit Smoking for at least 3 months in last 12 months**		**Quit Smoking for at least 6 months in last 12 months**		**Quit Attempts in last 12 months**			
**Overall quit rate (%)**	4.3	(3.5–5.2)	4.2	(3.4–5.1)	3.5	(2.8–4.4)	2.6	(2.0–3.4)	36.7	(34.0–39.4)		
**Adjusted odds ratio**												
**Household Wealth **												
Middle vs Low	0.85	(0.48–1.51)	0.88	(0.49–1.60)	0.68	(0.35–1.30)	0.69	(0.32–1.52)	1.19	(0.94–1.50)		
High vs Low	1.62	(0.89–2.95)	1.72	(0.92–3.19)	1.38	(0.72–2.63)	1.14	(0.53–2.46)	1.15	(0.89–1.49)		
**Education**												
Middle vs Low	2.03	(1.21–3.39)	1.98	(1.17–3.36)	1.76	(0.99–3.15)	2.05	(1.02–4.16)	1.16	(0.89–1.53)		
High vs Low	1.45	(0.68–3.09)	1.43	(0.66–3.09)	1.27	(0.57–2.82)	1.47	(0.55–3.97)	1.19	(0.85–1.68)		
**Residence**												
Urban vs Rural	1.11	(0.77–1.61)	1.10	(0.75–1.61)	1.32	(0.87–2.01)	1.20	(0.73–1.97)	1.30	(1.06–1.59)		
**Employment Status**												
Employed vs Not Employed	1.30	(0.73–2.32)	1.30	(0.72–2.33)	1.18	(0.64–2.19)	1.17	(0.55–2.47)	1.27	(0.93–1.75)		
**ITC**	**Quit Smoking in last 12 months**		**Quit Smoking for at least 1 month in last 12 months**		**Quit Smoking for at least 3 months in last 12 months**		**Quit Smoking for at least 6 months in last 12 months**		**Quit Attempts in last 12 months**			
**Overall quit rate (%)**	7.8	(5.8–10.2)	7.6	(5.6–9.9)	5.7	(4.4–7.2)	4.1	(3.0–5.5)	73.9	(68.8–78.5)		
**Adjusted odds ratio**												
**Household Income**												
Middle vs Low	0.75	(0.41–1.38)	0.57	(0.29–1.11)	0.83	(0.35–1.95)	0.62	(0.22–1.71)	0.55	(0.37–0.80)		
High vs Low	0.55	(0.28–1.06)	0.58	(0.09–3.93)	0.51	(0.22–1.18)	0.46	(0.19–1.11)	0.55	(0.37–0.81)		
**Education**												
Middle vs Low	0.84	(0.37–1.94)	0.81	(0.35–1.90)	1.05	(0.39–2.84)	0.97	(0.39–2.43)	1.35	(0.82–2.23)		
High vs Low	1.09	(0.36–3.30)	1.07	(0.35–3.24)	1.50	(0.49–4.61)	1.68	(0.51–5.58)	1.19	(0.60–2.38)		
**Residence**												
Urban vs Rural	0.73	(0.42–1.25)	0.72	(0.42–1.24)	0.57	(0.32–1.01)	0.61	(0.32–1.17)	0.54	(0.33–0.89)		
**Employment Status**												
Employed vs Not Employed	0.96	(0.55–1.66)	0.93	(0.54–1.59)	0.86	(0.44–1.64)	0.58	(0.31–1.11)	1.02	(0.56–1.84)		
**URUGUAY**												
**GATS**	**Quit Smoking in last 12 months**		**Quit Smoking for at least 1 month in last 12 months**		**Quit Smoking for at least 3 months in last 12 months**		**Quit Smoking for at least 6 months in last 12 months**		**Quit Attempts in last 12 months**			
**Overall quit rate (%)**	11.9	(9.7–14.5)	11.4	(9.2–14.0)	9.9	(7.8–12.5)	7.6	(5.7–10.0)	48.6	(45.0–52.3)		
**Adjusted odds ratio**												
**Household Wealth **												
Middle vs Low	1.61	(1.00–2.60)	1.64	(0.99–2.73)	1.41	(0.80–2.48)	1.90	(0.99–3.65)	0.72	(0.52–0.99)		
High vs Low	2.77	(1.61–4.79)	2.93	(1.67–5.13)	2.80	(1.56–5.02)	3.32	(1.73–6.36)	0.77	(0.53–1.13)		
**Education**												
Middle vs Low	0.74	(0.43–1.29)	0.70	(0.40–1.23)	0.67	(0.37–1.21)	0.72	(0.37–1.40)	0.89	(0.62–1.29)		
High vs Low	0.55	(0.19–1.59)	0.50	(0.16–1.55)	0.59	(0.19–1.84)	0.44	(0.13–1.47)	0.77	(0.42–1.43)		
**Residence**												
Urban vs Rural	0.58	(0.38–0.88)	0.59	(0.38–0.93)	0.61	(0.37–0.99)	0.59	(0.34–1.01)	0.91	(0.68–1.22)		
**Employment Status**												
Employed vs Not Employed	0.78	(0.50–1.22)	0.78	(0.49–1.23)	0.78	(0.52–1.19)	0.73	(0.46–1.18)	1.05	(0.78–1.41)		
**ITC**	**Quit Smoking in last 12 months**		**Quit Smoking for at least 1 month in last 12 months**		**Quit Smoking for at least 3 months in last 12 months**		**Quit Smoking for at least 6 months in last 12 months**		**Quit Attempts in last 12 months**			
**Overall quit rate (%)**	9.4	(7.1–12.2)	8.9	(6.6–11.7)	7.1	(5.2–9.5)	4.4	(2.9–6.4)	40.8	(35.2–46.5)		
**Adjusted odds ratio**												
**Household Income**												
Middle vs Low	0.81	(0.40–1.66)	0.89	(0.44–1.82)	0.99	(0.47–2.07)	0.87	(0.37–2.04)	0.67	(0.40–1.14)		
High vs Low	3.99	(1.54–10.35)	4.28	(1.64–11.13)	2.36	(0.88–6.30)	1.74	(0.47–6.50)	1.23	(0.51–3.12)		
**Education**												
Middle vs Low	1.99	(0.90–4.40)	1.77	(0.78–4.01)	1.46	(0.67–3.19)	1.16	(0.45–3.00)	1.57	(0.90–2.74)		
High vs Low	1.11	(0.45–2.73)	0.96	(0.40–2.28)	0.82	(0.34–1.98)	0.73	(0.23–2.33)	1.09	(0.59–2.02)		
**Residence**												
Urban vs Rural	NA		NA		NA		NA		NA			
**Employment Status**												
Employed vs Not Employed	0.75	(0.37–1.49)	0.72	(0.36–1.43)	0.72	(0.34–1.56)	0.75	(0.27–2.10)	1.46	(0.90–2.39)		

Note: 95% confidence intervals are in parentheses. NA = Not available.

While the overall quit rates for each country do not necessarily conform across surveys, the AORs (shown in Table 2) tend to be in accord. It is reflected in the I-squared values with p-values greater than 0.10 based on the heterogeneity of effect size across surveys for each country across all cessation measures (see p-values of the test of heterogeneity in S6 Tables). The only exception observed was India, where the AORs for high versus low education differed significantly between GATS and ITC across all cessation measures.

Based on the pooled AORs by country and data source reported in Table 3, we found limited evidence of higher SES as measured by household income/wealth and education predicting higher odds of quitting (AOR>1). In contrast, there is limited but not statistically significant evidence of higher SES in terms of residence (urban) predicting lower odds of quitting (AOR<1). The non-employment status of smokers was the only SES measure that predicted successful quitting, with statistically significant AORs regardless of which measures of cessation was used, and regardless of data sources for some cessation measures. As Table 3 shows, pooled AORs for all durations of quitting from the two surveys centred around a value of 0.80 for employed smokers. This result suggests that employed smokers have around 20% lower odds of quitting than do smokers who are not employed.

**Table 3:** Pooled adjusted odds ratios (AOR) of quit rates by different indicators of socio-economic status, duration of abstinence and surveys.

**Comparison of socio-economic status**	**Survey**	**Quit smoking in last 12 months**			**Quit smoking for at least 1 month in last 12 months**			**Quit smoking for at least 3 months in last 12 months**		
		**Pooled AOR**	**95% CI**	**P-value of the test of AOR = 1**	**Pooled AOR**	**95% CI**	**P-value of the test of AOR = 1**	**Pooled AOR**	**95% CI**	**P-value of the test of AOR = 1**
**Wealth/Income**	** **	** **	** **	** **	** **	** **	** **	** **	** **	** **
**High vs Low Wealth/Income**	**GATS**	1.29	(0.77–2.17)	0.338	1.23	(0.74–2.03)	0.425	1.26	(0.78–2.01)	0.344
	**ITC**	1.27	(0.88–1.84)	0.196	1.21	(0.84–1.75)	0.314	1.22	(0.84–1.76)	0.293
	**Pooled**	1.28	(0.95–1.73)	0.110	1.22	(0.91–1.65)	0.187	1.24	(0.92–1.66)	0.155
**Middle vs Low Wealth/Income**	**GATS**	1.10	(0.88–1.39)	0.410	1.10	(0.85–1.43)	0.459	1.10	(0.82–1.49)	0.515
	**ITC**	1.11	(0.87–1.41)	0.423	1.01	(0.78–1.32)	0.929	1.06	(0.78–1.43)	0.725
	**Pooled**	1.11	(0.95–1.30)	0.207	1.08	(0.92–1.27)	0.350	1.10	(0.90–1.34)	0.372
**Education**	** **									
**High vs low education**	**GATS**	**1.33**	(0.95–1.87)	0.100	**1.45**	(1.08–1.95)	0.013	**1.43**	(1.07–1.92)	0.016
	**ITC**	1.02	(0.67–1.56)	0.925	1.17	(0.85–1.62)	0.330	1.18	(0.85–1.65)	0.329
	**Pooled**	1.15	(0.88–1.51)	0.313	**1.34**	(1.09–1.65)	0.005	**1.32**	(1.06–1.64)	0.014
**Middle vs low education**	**GATS**	1.02	(0.74–1.40)	0.916	1.01	(0.74–1.38)	0.960	1.07	(0.79–1.43)	0.672
	**ITC**	0.92	(0.73–1.16)	0.467	0.97	(0.74–1.27)	0.822	0.96	(0.72–1.29)	0.794
	**Pooled**	0.99	(0.81–1.22)	0.957	1.01	(0.83–1.24)	0.905	1.05	(0.86–1.27)	0.633
**Residence**	** **									
**Urban vs Rural**	**GATS**	0.89	(0.70–1.12)	0.326	0.86	(0.71–1.05)	0.137	0.89	(0.74–1.07)	0.213
	**ITC**	0.95	(0.56–1.59)	0.830	1.03	(0.61–1.74)	0.913	1.14	(0.53–2.44)	0.733
	**Pooled**	0.91	(0.74–1.12)	0.376	0.91	(0.75–1.10)	0.332	0.95	(0.76–1.20)	0.689
**Employment**	** **									
**Employed vs Not Employed**	**GATS**	0.73	(0.49–1.08)	0.113	0.75	(0.52–1.09)	0.128	**0.77**	(0.60–0.98)	0.037
	**ITC**	0.86	(0.71–1.05)	0.140	0.83	(0.67–1.04)	0.113	**0.79**	(0.62–1.01)	0.055
	**Pooled**	**0.82**	(0.67–0.99)	0.044	**0.80**	(0.66–0.98)	0.034	**0.78**	(0.66–0.92)	0.003
**Comparison of socio-economic status**	**Survey**	**Quit smoking for at least 6 months in last 12 months**			**Quit tobacco (both smoking and smokeless tobacco) in last 12 months**			**Quit attempt in last 12 months**		
		**Pooled AOR**	**95% CI**	**P-value of the test of AOR = 1**	**Pooled AOR**	**95% CI**	**P-value of the test of AOR = 1**	**Pooled AOR**	**95% CI**	**P-value of the test of AOR = 1**
**Wealth/Income**	** **	** **	** **	** **	** **	** **	** **	** **	** **	** **
**High vs Low Wealth/Income**	**GATS**	1.39	(0.90–2.14)	0.141	1.36	(0.42–4.42)	0.612	1.06	(0.90–1.24)	0.480
	**ITC**	1.31	(0.83–2.07)	0.241	1.41	(0.54–3.88)	0.470	1.10	(0.79–1.52)	0.583
	**Pooled**	**1.35**	(1.00–1.82)	0.053	1.25	(0.72–2.17)	0.434	1.07	(0.91–1.26)	0.433
**Middle vs Low Wealth/Income**	**GATS**	**1.32**	(0.95–1.82)	0.096	**1.57**	(1.04–2.37)	0.033	1.03	(0.90–1.18)	0.664
	**ITC**	1.02	(0.68–1.51)	0.941	1.18	(0.65–2.16)	0.585	0.93	(0.70–1.24)	0.623
	**Pooled**	1.18	(0.92–1.52)	0.202	**1.43**	(1.02–2.01)	0.039	0.98	(0.85–1.13)	0.801
**Education**	** **									
**High vs low education**	**GATS**	**1.34**	(0.96–1.88)	0.086	1.57	(0.33–7.57)	0.575	1.03	(0.84–1.25)	0.796
	**ITC**	1.19	(0.80–1.77)	0.392	0.82	(0.36–1.88)	0.636	1.14	(0.95–1.36)	0.150
	**Pooled**	**1.28**	(0.99–1.65)	0.063	1.13	(0.47–2.70)	0.782	1.07	(0.94–1.21)	0.332
**Middle vs low education**	**GATS**	0.99	(0.69–1.42)	0.947	0.93	(0.29–2.95)	0.902	1.05	(0.96–1.16)	0.279
	**ITC**	0.85	(0.60–1.20)	0.346	0.88	(0.49–1.56)	0.654	1.11	(0.95–1.30)	0.173
	**Pooled**	0.95	(0.74–1.21)	0.666	1.00	(0.61–1.63)	0.987	1.07	(0.99–1.16)	0.104
**Residence**	** **									
**Urban vs Rural**	**GATS**	0.91	(0.76–1.09)	0.313	1.27	(0.62–2.60)	0.518	1.01	(0.86–1.18)	0.920
	**ITC**	1.01	(0.58–1.74)	0.978	0.84	(0.46–1.54)	0.567	0.67	(0.41–1.10)	0.114
	**Pooled**	0.92	(0.77–1.09)	0.305	1.05	(0.72–1.52)	0.808	0.92	(0.77–1.08)	0.298
**Employment**	** **									
**Employed vs Not Employed**	**GATS**	**0.71**	(0.53–0.94)	0.018	0.85	(0.48–1.51)	0.585	0.93	(0.81–1.07)	0.335
	**ITC**	0.80	(0.58–1.12)	0.199	**0.67**	(0.43–1.05)	0.078	**1.12**	(0.94–1.33)	0.194
	**Pooled**	**0.75**	(0.61–0.93)	0.007	**0.75**	(0.54–1.05)	0.089	1.01	(0.90–1.14)	0.846

Note: The pooled AORS for quit tobacco (both smoking and smokeless tobacco) in the past 12 months are based on estimates from Bangladesh and India only. Other measures include all the eight countries. The AORs in bold are significant at 10% level.

## Discussion

Based on the analysis of eight LMICs, that had conducted GATS and the ITC survey during the same year, this study provides limited evidence to support the hypothesis that the probability of successful quitting is greater for smokers with higher SES as defined by household income, wealth and education. However, with respect to employment, the findings indicate that smokers without employment (e.g. students, homemakers, retirees, and the unemployed) have greater probability of successful quitting. Abdullah and Yam (2005) similarly observed that being in the “student/retired/others” category was associated with quitting among Hong Kong Chinese smokers [38].

The evidence that smokers who are not employed are more likely to quit than their employed counterparts seems counterintuitive from the perspective of environmental pressure to not smoke. Non-employed smokers are not subject to smoke-free policies in workplaces [52]. They do not face the social pressure not to smoke that working individuals tend to face from their co-workers. It is unlikely the case that smoking bans are more widely adopted and better enforced in other venues including homes than in workplaces in LMICs.

It is also unlikely that the employment status of individuals would be sensitive enough to capture the effect of household wealth on individuals’ decision to smoke. Wealth index is an indicator of household level economic status, which is accumulated over time. On the other hand, employment status is indicative of current individual economic status, which is inherently more transitory than household wealth. However, for students or homemakers, employment status is not necessarily reflective of household income status.

Students, who are predominantly younger adults, have shorter smoking histories and lower nicotine dependence. They are, therefore, more likely to succeed in quitting [53]. While quitting at any age may bring forth significant immediate health gain, the long-term benefit (e.g., gain in life expectancy) is even greater when smokers quit young [54]. The finding that non-employed students are more likely to quit is therefore positive from public health perspective. The higher success rate of quitting among homemakers is also expected to lead to added health gain from reduction in second-hand smoking among nonsmokers at home, both adults and children. Homemakers are traditionally women and have lower smoking prevalence than men. Why homemakers are more likely to quit is, however, unknown from existing literature.

Individual employment status correlates with household income only when the non-employed status refers to the unemployed or the retirees. Lack of employment and thus lack of earning can create stronger motivation to quit among the unemployed or the retirees mediating through loss of affordability of all products including tobacco. The unemployed or the retirees in many LMICs may not have access to strong social insurance mechanisms (e.g., unemployment/retirement benefit programs) needed to weather any difficulties created by the lack of income. In such settings, lack of employment means significant drop in household income, increase in vulnerability and decrease in overall affordability for this group. Affordability can, however, be reduced by either loss of income or increase in prices or a combination of the two. From the perspective of tobacco control and public health, raising tobacco product prices induced by tax increases has proven to be an effective measure that can reduce affordability of tobacco products, encourage cessation and reduce tobacco consumption [2, 45, 53]. The finding in this paper that affordability matters in smokers’ decision to quit is in accord with this evidence.

The mixed evidence from the LMICs observed in this paper suggests that the explanations of lower quitting probability among lower SES smokers from other studies in high-income countries may not necessarily generalize to LMICs. Uruguay is the only country in this study that was transitioning to high-income status at the time of the survey, and is the only country where both GATS and ITC indicated a positive socio-economic gradient of quit rate with respect to high versus low household income/wealth. It follows that the association between quitting behaviour and socio-economic predictors must be considered in the context of the country’s income status. It is also important to consider the stage of the tobacco epidemic, the strength of the tobacco control policy environment, the variation in socio-economic and cultural contexts, and the sensitivity of low-SES smokers to tobacco control policies compared to high-SES smokers in a specific country setting.

If a country is in the advanced stage of the tobacco epidemic, when the smoking-attributable mortality reaches its peak and smoking prevalence is declining, quitting would generally be significantly higher among the higher-educated and higher-SES strata. Because higher-SES people are more sensitive to the dissemination of knowledge and more aware of the health harms of smoking [55–57]. This may not necessarily be the case in LMICs in the earlier stages of the epidemic [58]. In a recent systematic review, Casetta and colleagues similarly concluded that differences in tobacco consumption between income groups are more marked in high-income countries, which may be due to these countries’ ability to implement tobacco control policies and achieve greater awareness of associated health risks among their high-income populations [59]. Thus, tobacco control strategies adopted by a country may account for the socio-economic gradient of quitting behaviour as well. For example, in a country that is actively pursuing tobacco tax and price policy, low-SES individuals, who are more responsive to tax and price increases, could be more successful in quitting tobacco use. Based on a duration analysis of smoking initiation and quitting behaviour of a sample of Irish women, Madden (2007) concluded that taxation is most effective in inducing quitting among those with the lowest level of education [60]. Although tax increases can impose a significant financial burden on low-income smokers [61], of all the available tobacco control interventions, only increases in tobacco prices have been clearly shown to reduce socio-economic disparities in smoking and consequent health outcomes [62, 63].

The present study has some limitations. First, we did not examine the interaction between the SES indicators and tobacco control policy variables that can potentially influence the socio-economic gradient of quitting probability. For example, an increase in the cost of purchasing tobacco products driven by higher tax and price is more likely to trigger quitting among low-SES smokers [64, 65]. We also did not examine relevant smoking-related variables as mediators of the association between SES indicators and quitting behaviour, something that should be considered in future studies.

Second, tertiles of SES based on household income variable in ITC data and wealth index in GATS data are not identical. Current household income is a measure of current SES while wealth reflects permanent SES of a household. Although we have taken great care to make the classification of income, wealth and education consistent across countries and data sources, any unintended misclassification may partly contribute to the inconsistent effects of income, wealth and education on quitting behaviour.

A third limitation lies in the differential classification of quitters in the two surveys: the ITC surveys included former cigarette smokers only, while GATS included former smokers of all combustible tobacco products including cigarettes. The design of the GATS questionnaire does not allow the analysis to identify only cigarette smokers in calculating quit rates. Although factory-made cigarettes account for approximately 90% of combustible tobacco products worldwide, use of other combustible tobacco products is significant in some countries, such as bidi in Bangladesh and India and RYO in Thailand [66]. Bidi, a much cheaper product than cigarettes, is commonly consumed by poorer people [67]. In such settings, cigarette price increases may not effectively lead to quitting smoking, as smokers may simply switch to the cheaper alternatives [29, 68]. Limiting the analysis to only former cigarette smokers may therefore bias the estimates for these countries. Considering the high level of non-cigarette combustible products use in Bangladesh, India and Thailand, we identified smokers of all products in these three countries. The data on non-cigarette smoked tobacco products were not reported in the other five countries.

Fourth, the status of tobacco use and quitting of respondents is based on self-reported and retrospective data. These data are often subject to recall bias, social desirability bias and other measurement errors. These might vary systematically among countries to bias country-specific estimates.

Fifth, the ITC surveys provide nationally representative data for only three (i.e. Bangladesh, Malaysia, Thailand) of the eight countries, with the rest limited to certain states (e.g., India) or cities (e.g., Brazil, China, Mexico and Uruguay). To overcome the potential limitation of lack of national representativeness of the ITC surveys, we employed GATS, as a second source of data where available for the countries under study with approximately contemporaneous data collection as with ITC.

Finally, this study does not provide conclusive evidence on the role of socio-economic disparities in cessation behaviour in explaining the disparities in smoking in LMICs. It leads us to the next question: Do socio-economic disparities in initiation of smoking contribute to the socio-economic inequality in smoking? It remains to be answered in future research.

## Conclusion

Lack of clear evidence of socio-economic inequality in adult cessation behaviour in LMICs suggests that lower-SES smokers are not less successful in their attempts to quit than their higher-SES counterparts. Specifically, lack of employment, which is typically indicative of younger age and lower nicotine dependence for students, or lower personal disposable income and lower affordability for the unemployed and the retired, may be associated with quitting. Raising taxes and prices of tobacco products that reduces affordability of tobacco products might be a key strategy for inducing cessation behaviour among current smokers and reducing overall tobacco consumption. Because low-SES smokers are more sensitive to price increases, tobacco taxation policy can induce disproportionately larger decreases in tobacco consumption among them and help reduce socio-economic disparities in smoking and consequent health outcomes.

## Supporting information

S1 Tables (DOCX)

S2 Tables (XLSX)

S3 Tables (XLSX)

S4 Tables (XLSX)

S5 Tables (XLSX)

S6 Tables (XLSX)
