# Supplementary material for: Socioeconomic patterns of smoking cessation behavior in low and middle-income countries: Emerging evidence from the Global Adult Tobacco Surveys and International Tobacco Control Surveys
Source: PLoS One. 2019 Sep 6;14(9):e0220223. doi: 10.1371/journal.pone.0220223 (PMC6730869; doi:10.1371/journal.pone.0220223)
Supplement: S1 Tables — (DOCX) [file pone.0220223.s001.docx]

**Table A1: Waves and income status of countries in GATS and ITC surveys**

| Country | Income status | GATS | ITC |
| --- | --- | --- | --- |
| Bangladesh | Low | 2009 | Wave 1: 2009 |
|  |  |  | Wave 2: 2010 |
| Brazil | Upper-middle | 2008 | Wave 1: 2009 |
|  |  |  | Wave 2: 2012/2013 |
| China | Upper-middle | 2010 | Wave 3: 2009 |
|  |  |  | Wave 4: 2011/2012 |
| India | Lower-middle | 2009/2010 | Wave 1: 2010/2011 |
|  |  |  | Wave 2: 2012/2013 |
| Malaysia | Upper-middle | 2011 | Wave 4: 2009 |
|  |  |  | Wave 5: 2011/2012 |
| Mexico | Upper-middle | 2009 | Wave 3: 2008 |
|  |  |  | Wave 4: 2010 |
| Thailand | Lower-middle | 2011 | Wave 4: 2009 |
|  |  |  | Wave 5: 2011 |
| Uruguay | Upper-middle | 2009 | Wave 2: 2008/2009 |
|  |  |  | Wave 3: 2010/2011 |

**Table A2: The classification of educational status by country in ITC and GATS data.**

| Country | Low | Middle | High |
| --- | --- | --- | --- |
| Bangladesh | Illiterate | Primary (1-5 years) | SSC (9-10 years) |
|  |  | Secondary (6-8 years) | HSC (11-12 years) |
|  |  |  | Bachelor's degree (14-16 years) |
|  |  |  | Master's degree (15-17 years) |
|  |  |  | Above Master's degree |
| Brazil | Never attended school | High school | University degree (1-8 years) |
|  | Primary (1-4 years) |  | Post graduate |
|  | Secondary (1-4 years) |  |  |
|  | Admission |  |  |
|  | basic (1-8 years) |  |  |
| China | No education | Junior high school | College |
|  | Elementary school | High school, technical | University or higher |
|  |  | high school |  |
| India | Illiterate | Secondary school | Graduate (BA/BSc/Diploma) |
|  | Literate, no formal |  | Post graduate/professional |
|  | education |  | Above post graduate (PhD) |
|  | Up to primary school |  |  |
|  | Middle school |  |  |
| Malaysia | No schooling | Lower secondary | Bachelor's degree |
|  | Lower elementary | Upper secondary | Higher than Bachelor's degree |
|  | Upper elementary | Pre-university |  |
|  |  | Diploma/certificate |  |
| Mexico | No Schooling/incomplete | Middle school complete | University (incomplete) |
|  | Elementary school | Technical/vocational | University (complete) |
|  | Primary school complete | school complete | Post graduate |
|  |  | High school complete |  |
| Thailand | No schooling | lower secondary | diploma/certificate |
|  | Lower than elementary | upper secondary | Bachelor's degree |
|  | Elementary |  | higher than Bachelor's |
| Uruguay | Elementary | Technical school | Army/police/others |
|  | Middle school completed | High school (incomplete | teachers/trainers |
|  |  | or completed) | University |

**Table A3: The classification of income by country in ITC data.**

| Country | Type | Low | Middle | High |
| --- | --- | --- | --- | --- |
| Bangladesh | monthly | < 5,000 taka | 5000 to < 10,000 taka | 10,000 to < 15,000 taka |
|  |  |  |  | 15,000 to < 20,000 taka |
|  |  |  |  | 20,000+ taka |
| Brazil | monthly | no income | R$1245 to < R$2075 | R$4150 to < R$8350 |
|  |  | < R$415 | R$2075 to < R$4150 | R$8350 to < R$12,450 |
|  |  | R$415 to < R$830 |  | R$12,450 to < R$16,700 |
|  |  | R$830 to < R$1245 |  | R$16,700 to < R$20,750 |
|  |  |  |  | >= R$20,750 |
| China | monthly | < 1000 Yuan | 1000 to 2999 Yuan | 3000 to 4999 Yuan |
|  |  |  |  | 5000 to 6999 Yuan |
|  |  |  |  | 7000 to 8999 Yuan |
|  |  |  |  | >= 9000 Yuan |
| India | monthly | < 5000 rupees | 5000 to < 10,000 | 15,000 to < 20,000 |
|  |  |  | 10,000 to < 15,000 | >= 20,000 |
| Malaysia | annual | *Income was collected as a continuous measure. A per capita measure was created for each respondent by dividing household income by the number of adults in the household. This measure was then split into tertiles to create the low/middle/high income categories. A fourth not stated category was included for respondents not reporting income.* | | |
| Mexico | monthly | <= $1500 | $3001 to $5000 | $5001 to $8000 |
|  |  | $1501 to $3000 |  | $8001 to $10,000 |
|  |  |  |  | $10,001 to $15,000 |
|  |  |  |  | > $15,000 |
| Thailand | annual | *Same procedure as Malaysia* | | |
| Uruguay | monthly | <= $4500 | $8001 to $12,500 | $30,001 to $40,000 |
|  |  | $4501 to $8000 | $12,501 to $15,000 | $40,001 to $50,000 |
|  |  |  | $15,001 to $20,000 | $50,001 to $60,000 |
|  |  |  | $20,001 to $30,000 | $60,001 to $70,000 |
|  |  |  |  | $70,001 to $80,000 |
|  |  |  |  | > $80,000 |
|  |  |  |  |  |
